# Supplementary material for: A Flexible Method for Nanofiber-based 3D Microfluidic Device Fabrication for Water Quality Monitoring
Source: Micromachines (Basel). 2020 Mar 6;11(3):276. doi: 10.3390/mi11030276 (PMC7143371; doi:10.3390/mi11030276)
Supplement: Supplementary file 1 [file micromachines-11-00276-s001.zip › micromachines-738405 -sup-proof/Support material/Support material-738405 -sup-proofreading.docx]

Supplementary Materials: A Flexible Method for Nanofiber-based 3D Microfluidic Devices Fabrication for Water Quality Monitoring

Xiaojun Chen, Deyun Mo and Manfeng Gong


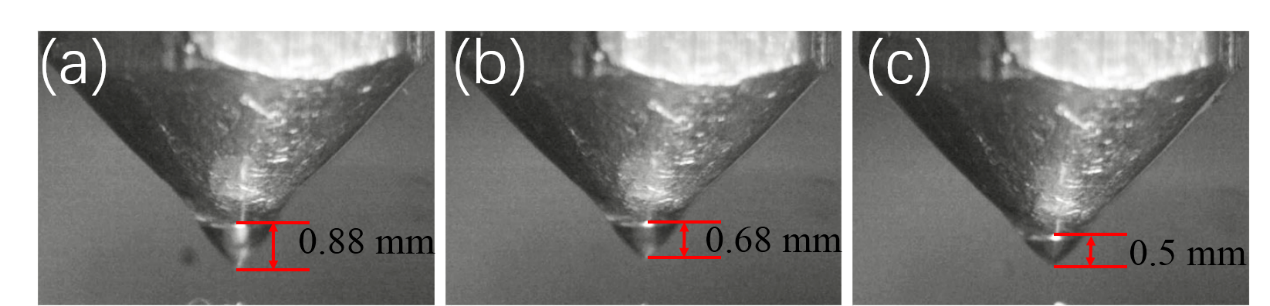


**Figure S1.** Images of Taylor cones at different voltages. (**a**) 4 kV; (**b**) 5 kV; (**c**) 6 kV. Collecting distance, 3 mm; heating temperature, 95 °C.

**Table S1.** Wax Viscosity and Surface Tension at Different Temperatures.

| Temperatures (°C) | 70 | 75 | 80 | 85 | 90 | 95 | 100 |
| --- | --- | --- | --- | --- | --- | --- | --- |
| Viscosity（cP） | 33.65 | 30.24 | 28.51 | 25 | 23.44 | 22.46 | 21.1 |
| Surface tension（mN/m） | 30.3 | 29.95 | 29.6 | 29.25 | 28.9 | 28.55 | 28.2 |


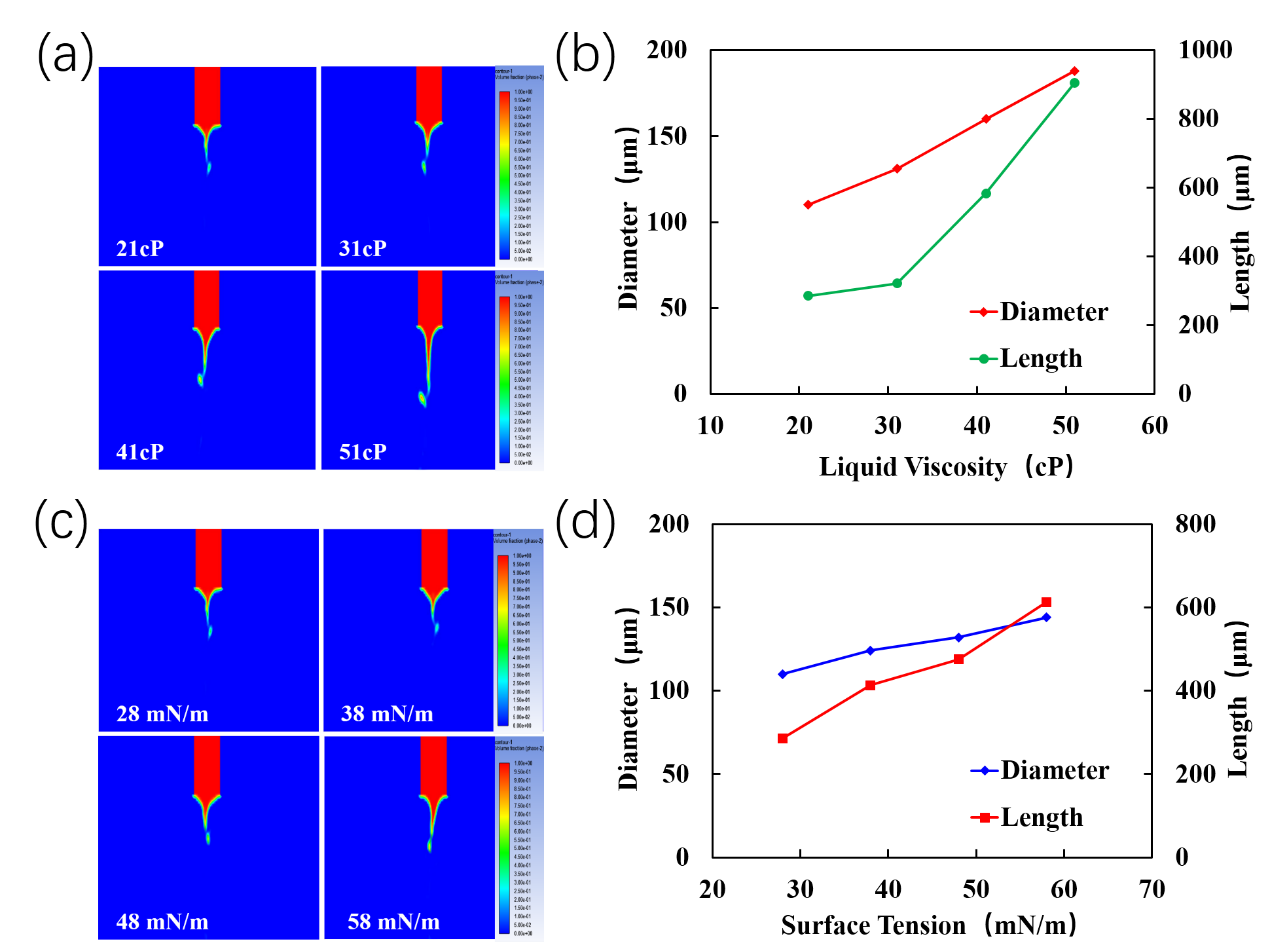


**Figure S2.** Simulation of the effects of fluid viscosity and surface tension on the Taylor cone shape of the droplet. (**a**) The liquid viscosity range from 21 cP to 51 cP, V = 5 kV, t = 1.2 s; (**b**) Relationship between droplet diameter and Taylor cone length under different liquid viscosity; (**c**) The liquid surface tension range from 28 mN/m to 58 mN/m, V = 5 kV, t = 0.8 s; (**d**) Relationship between droplet diameter and Taylor cone length under different surface tension.

**Table S2.** Resolution and cost of each method.

| **Method** | **Material** | **Resolution (μm)** | **Features** |
| --- | --- | --- | --- |
| Wax printing | Wax | 561 ± 45 | Low-cost, simple fabricate, mass production; low resolution |
| Photolithography | SU8-2010  OTS | 186 ± 13  233 ± 30 | High resolution; expensive equipment, complex steps and expensive reagents |
| Inkjet printing | AKD | ~590 | Low-cost, cheap reagents, mass production; need to use a printer |
| Screen printing | Wax | 650 ± 71 | Low-cost, simple steps, mass production; low resolution, different pattern need different printing wire |
| Laser-based | Light-sensitive polymer | ~80 | High resolution, complex pattern; expensive, complicated to operate |
| Plasma treatment | Fluorocarbon | 300–500 | High-cost, one-step, all-dry; need two stainless steel, masks |
| Our work | Wax | ~296 | Low-cost, high resolution, simple fabricate and one-step prototype; slow flow rate |


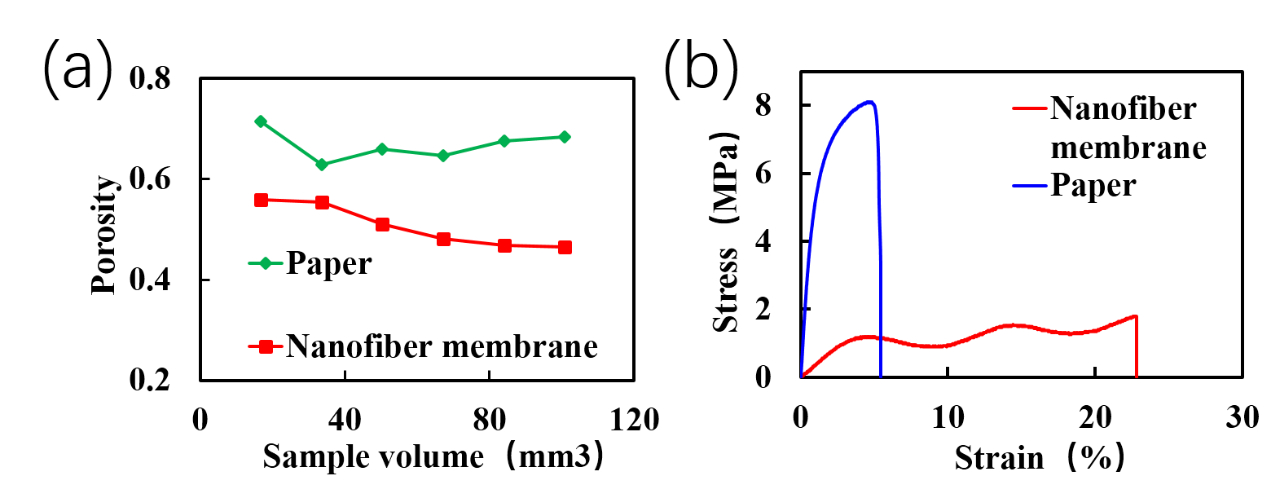


**Figure S3.** Comparison of performance of filter paper and nanofiber membrane. (**a**) Relationship of sample volume and porosity; Data shown are average of six parallel experiments. (**b**) Relationship of sample stress and strain at tensile rate of 20 mm/min.


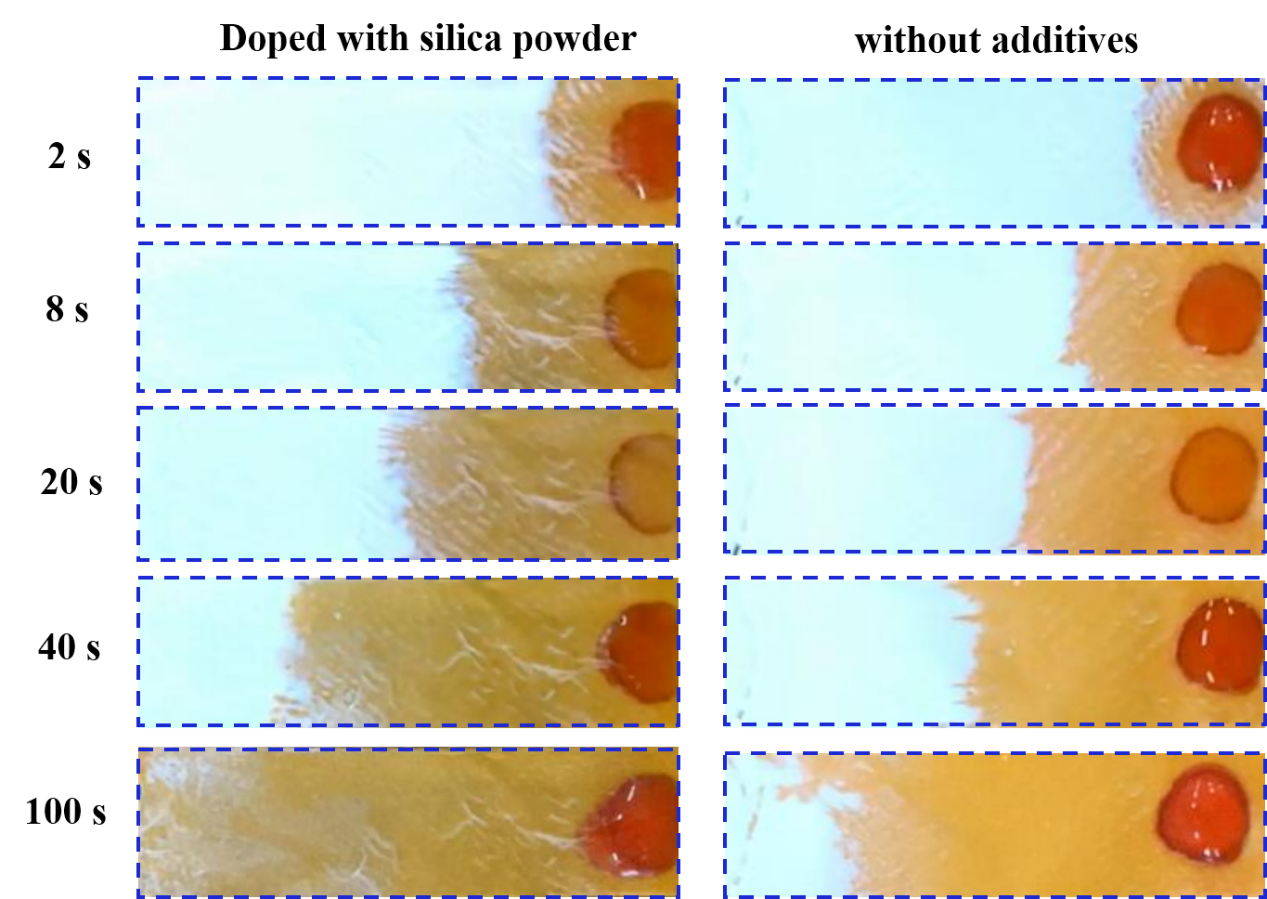


**Figure S4.** Capillary flow of nanofiber membranes doped with silica powder and without silica powder ranged from 2 s to 100 s.

**Video S1:** Patterning of hydrophobic barriers by electrostatic wax printing.

Electric field voltage, 5 kV; nozzle heating temperature: 100 °C; nozzle-to-substrate distance, 3 mm.
